# Bioactive Terpenes from Marine-Derived Fungi

**DOI:** 10.3390/md13041966

**Published:** 2015-04-03

**Authors:** Ahmed M. Elissawy, Mohamed El-Shazly, Sherif S. Ebada, AbdelNasser B. Singab, Peter Proksch

**Affiliations:** 1 Department of Pharmacognosy and Phytochemistry, Faculty of Pharmacy, Ain-Shams University, Organization of African Unity Street 1, 11566 Cairo, Egypt; E-Mails: aelissawy@pharma.asu.edu.eg (A.M.E.); mohamed.elshazly@pharma.asu.edu.eg (M.E.-S.); sherif_elsayed@pharma.asu.edu.eg (S.S.E.); Dean@pharma.asu.edu.eg (A.B.S.); 2 Institut für Pharmazeutische Biologie und Biotechnologie, Heinrich-Heine Universität, Geb. 26.23, Universitätsstrasse 1, D-40225 Düsseldorf, Germany

**Keywords:** marine-derived fungi, secondary metabolites, terpenes, bioactivity

## Abstract

Marine-derived fungi continue to be a prolific source of secondary metabolites showing diverse bioactivities. Terpenoids from marine-derived fungi exhibit wide structural diversity including numerous compounds with pronounced biological activities. In this review, we survey the last five years’ reports on terpenoidal metabolites from marine-derived fungi with particular attention on those showing marked biological activities.

## 1. Introduction

Marine-derived fungi have proven to be a prolific source of secondary metabolites with interesting structural properties and biological activities [1,2,3,4]. In this review, we survey terpenes reported in literature from marine-derived fungi over the years from 2010 to 2014. The compounds covered include monoterpenes, sesquiterpenes, diterpenes, sesterterpenes, and triterpenes in addition to prenylated polyketides known as “meroterpenes”.

## 2. Different Classes of Terpenoids

### 2.1. Monoterpenes (C10)

**Figure 1:**
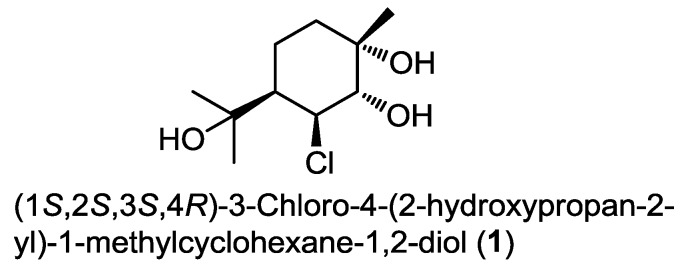
The only monoterpene derivative isolated from marine fungi since 2006.

Monoterpenes have been rarely isolated from marine-derived fungi over the last decade. To the best of our knowledge, the only example reported till now dates back to 2006 and it was identified as a chlorinated monoterpene derivative, (1*S*,2*S*,3*S*,4*R*)-3-chloro-4-(2-hydroxypropan-2-yl)-1-methylcyclohexane-1,2-diol (**1**) (Figure 1). It was obtained from the extract of a fermentation broth of the mangrove-derived endophytic fungus *Tryblidiopycnis* sp. isolated from *Kandelia* woody tissue in Hong Kong [5].

### 2.2. Sesquiterpenes (C15)

**Figure 2:**
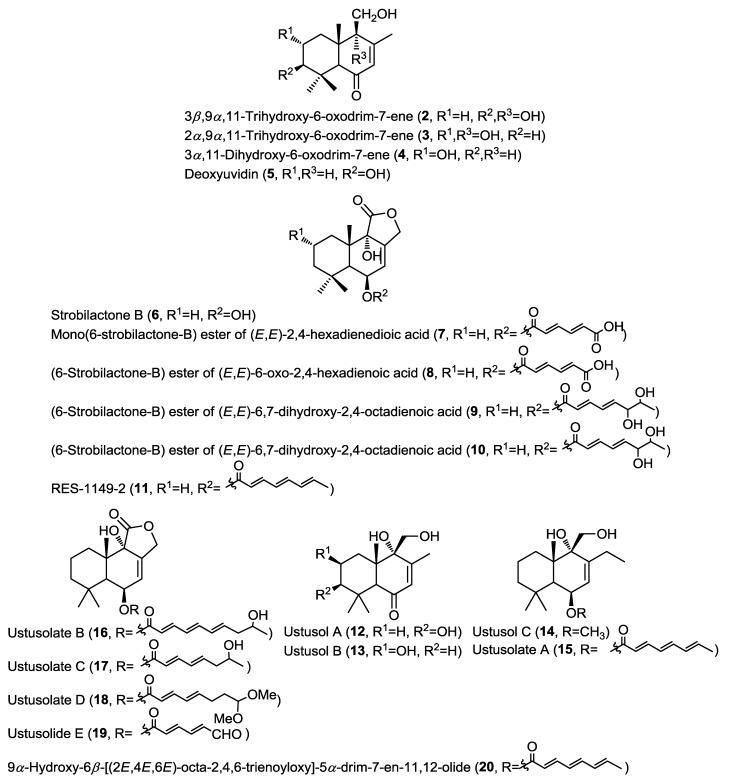
Sesquiterpenes isolated from *Aspergillus ustus* and *A. ustus*.

Purification of the cytotoxic ethyl acetate extract of the fermentation broth of the fungus *Aspergillus ustus* isolated from the marine sponge *Suberites domuncula* collected from the Adriatic Sea yielded new drimane-type sesquiterpenoids (**2**–**4** and **7**–**10**) together with the known related compounds deoxyuvidin B (**5**), strobilactone B (**6**) and RES-1149-2 (**11**) (Figure 2) [6]. Compounds **5** and **6** were previously reported from terrestrial fungi and this was the first report from a marine-derived fungus [7,8]. Compounds **2**–**5** were identified as hydroxylated derivatives of drim-7-ene-6-one, whereas compounds **7**–**11** were identified as esters of 6β,9α-dihydroxy-5α-drim-7-en-11,12-olide with polyunsaturated acid substituents at C-6. Cytotoxicity of compounds **2**–**10** has been evaluated against a panel of cancer cell lines, including mouse lymphoma (L5178Y), human cervical cancer (HeLa), and rat pheochromocytoma (PC12) cells. Results indicated that only compounds **7**, **8** and **10** showed cytotoxicity with compound **10** being the most active (IC_50_ = 1.6, 15.8, and 19.3 µM, respectively) and it exhibited a higher selectivity toward mouse lymphoma (L5178Y) cells. This activity was strongly related to the esterification with polyunsaturated acids at C-6.

Simultaneous report on a different isolate of the same fungus *A. ustus*, isolated from the rhizospheric soil of the mangrove plant *Bruguiera gymnorrhiza*, revealed the isolation of the structurally closely related drimane sesquiterpenoids ustusols A–C (**12**–**14**) and the ester derivatives ustusolates A–E (**15**–**19**) together with the known compound 9α-hydroxy-6β-[(2*E*,4*E*,6*E*)-octa-2,4,6-trienoyloxy]-5α-drim-7-en-11,12-olide (**20**) (Figure 2) [9]. New compounds were evaluated for their cytotoxic activity against A549 and HL-60 cancer cell lines. Only ustusolates C (**17**) and E (**19**) showed cytotoxicity against A549 and HL-60 cell lines with IC_50_ values of 10.5 and 9.0 µM, respectively, whereas ustusolate A (**14**) showed weak growth inhibition against both A549 and HL-60 cell lines with IC_50_ values of 30.0 and 20.6 µM, respectively. These results may indicate that the length and substituents on the unsaturated side chain can influence the cytotoxicity of the ustusolates.

**Figure 3:**
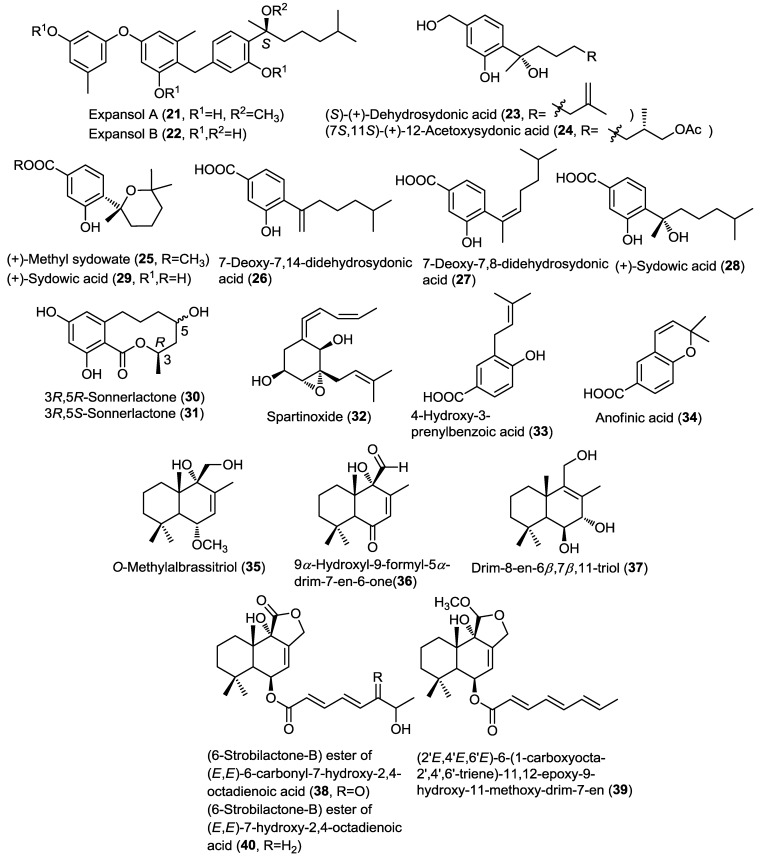
Sesquiterpenes isolated from *Penicillium expansum*, *Aspergillus* sp., unidentified endophytic fungal strain Zh6-B1, *Phaeosphaeria spartinae* and *Aspergillus ustus*.

Four new phenolic bisabolane sesquiterpenoids (**21**–**24**) were reported from the endophytic marine fungus *Penicillium expansum* 091006 isolated from the roots of the mangrove plant *Excoecaria agallocha* (China) (Figure 3) [10]. Expansols A (**21**) and B (**22**) contain a diphenyl ether moiety related to diorcinol linked with the phenolic bisabolane sesquiterpene through a methylene bridge, whereas (*S*)-(+)-11-dehydrosydonic acid (**23**) and (7*S*,11*S*)-(+)-12-acetoxysydonic acid (**24**) are derivatives of sydonic acid differing from the latter compound by unsaturation and acetylation, respectively. Bisabolane sesquiterpenoids are uncommon fungal metabolites and only a limited number of reports so far indicated their occurrence in fungi [11,12]. Moreover, this was the first report of phenolic bisabolane coupled with diphenyl ethers. Compounds **21**–**24** were subjected to cytotoxicity studies against A549 and HL-60 cancer cell lines. Results revealed that only expansol A (**21**) exhibited moderate cytotoxicity against the HL-60 cell line with IC_50_ of 15.7 µM, whereas, expansol B (**22**) inhibited the proliferation of A549 and HL-60 cells with IC_50_ values of 1.9 and 5.4 µM, respectively. These results highlighted the importance of coupling diphenyl ethers with the phenolic bisabolane sesquiterpenoids for enhancing their cytotoxic activity.

A simultaneous report indicated the isolation of three new structurally related bisabolane sesquiterpenoids (**25**–**27**) together with the known sesquiterpenes (+)-sydowic acid (**28**) and (+)-sydonic acid (**29**), from the fungus *Aspergillus* sp. cultured from the marine gorgonian *Dichotella gemmacea* (Figure 3) [13]. This was the first report of natural products from gorgonian-derived fungus. Methyl sydowate (**25**) is an ester of **28**, and the authors implemented HPLC to ensure that **25** is a genuine natural product and not an artifact. Similarly, 7-deoxy-7,14-didehydrosydonic acid (**26**), and 7-deoxy-7,8-didehydrosydonic acid (**27**) were confirmed as genuine natural products by treating the crude extract with mild acidic conditions which failed to provoke the formation of **26** and **27**. This is the first report for the isolation of (+)-sydowic acid and (+)-sydonic acid which were previously only known as (−) isomers. Antimicrobial evaluation of compounds **25**, **28** and **29** showed weak activity against *Staphylococcus aureus* and no activity against methicillin resistant *S. aureus* [13].

Two new isomeric sesquiterpene lactones, 3*R*,5*R*-sonnerlactone (**30**) and 3*R*,5*S*-sonnerlactone (**31**), were isolated from an unidentified endophytic fungal strain Zh6-B1 cultured from the bark of the mangrove plant *Sonneratia apetala* (Figure 3) [14]. The two compounds possess identical 1D NMR and MS spectra and the differentiation was achieved using X-ray crystallography of **30** and through comparing their NOESY spectra. Cytotoxicity investigation of both compounds was conducted on multi-drug resistant human oral floor carcinoma cell lines KV/MDR and revealed weak cytotoxic activity for both compounds [14].

Spartinoxide (**32**), a new sesquiterpene, was isolated from the fungus *Phaeosphaeria spartinae* cultured from the marine alga *Ceramium* sp. collected in North Sea, Germany, together with the known compounds 4-hydroxy-3-prenyl-benzoic acid (**33**) and anofinic acid (**34**) (Figure 3) [15]. Spartinoxide (**32**) is an optical isomer of the known fungal metabolite A82775C, having identical 1D, 2D and MS spectra whereas it differs in the optical rotation. Compounds **32**–**34** were investigated for their enzymatic inhibitory activity against a panel of human enzymes including human leukocyte elastase (HLE), trypsin, acetylcholinesterase and cholesterolesterase. Compounds **32** and **33** showed potent inhibitory activity against Human leukocyte elastase (HLE), responsible for inflammatory conditions such as pulmonary emphysema and cystic fibrosis [15].

A further report on the fungus *Aspergillus ustus* cultured from the rhizospheric soil of the mangrove plant *Acrostichum aureurm* yielded five new drimane-type sesquiterpenoids (**35**–**39**), *O*-methylalbrassitriol (**35**), 9α-hydroxyl-9-formyl-5α-drim-7-en-6-one (**36**), drim-8-en-6β,7β,11-triol (**37**), (6-strobilactone-B) ester of (*E*,*E*)-6-carbonyl-7-hydroxy-2,4-octadienoic acid (**38**), 11-methoxy-drim-7-ene (**39**) together with the known compound (6-strobilactone-B) ester of (*E*,*E*)-7-hydroxy-2,4-octadienoic acid (**40**) (Figure 3) [16]. Compounds **35**–**40** were investigated for their cytotoxic activity against a panel of cancer cell lines, and only **38** showed cytotoxic activity against P388 cell line with IC_50_ value of 8.7 µM, whereas other compounds exhibited no activity [16]. The difference in the cytotoxic activity between **38** and **40** hinted to the possible role of the carbonyl group at C-6' in the activity of compound **38**.

**Figure 4:**
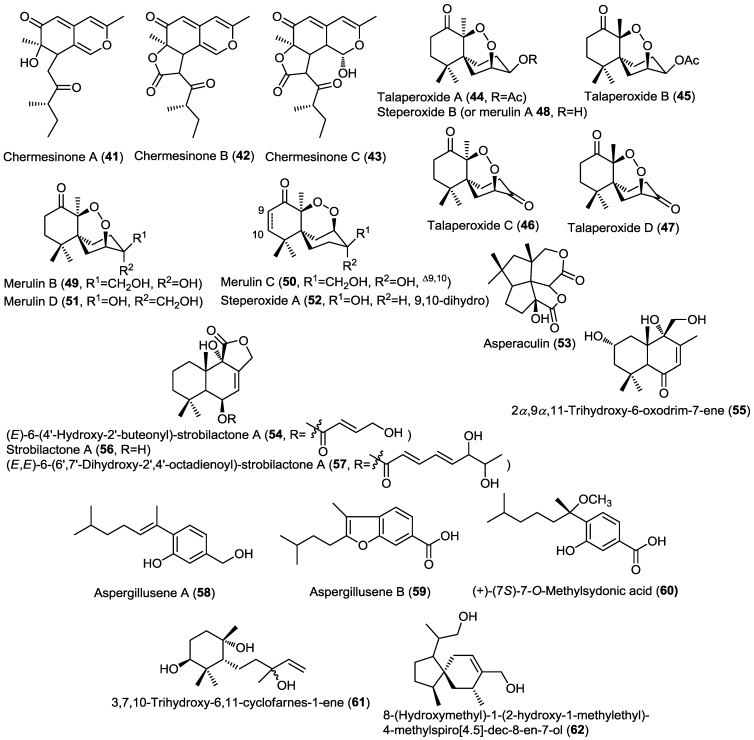
Sesquiterpenes isolated from *Penicillium chermesinum* (ZH4-E2), *Talaromyces flavus*, basidiomycete fungus XG8D, *Aspergillus aculeatus*, *Aspergillus insuetus* (OY-207), *Aspergillus sydowii* PSU-F154, and *Eutypella scoparia* FS26.

Three new azaphilone sesquiterpenoids, chermesinones A–C (**41**–**43**), were obtained from the endophytic fungus *Penicillium chermesinum* (ZH4-E2) isolated from the stems of the mangrove plant *Kandelia candel* (Figure 4) [17]. The absolute configuration of **41** was determined by X-ray crystallography. Compounds **41**–**43** were investigated for their inhibitory activity against α-glucosidase and acetylcholinesterase enzymes, and only chermesinone A (**41**) showed mild α-glucosidase inhibitory activity.

Four new norsesquiterpene peroxides, talaperoxides A–D (**44**–**47**), were isolated from the endophytic fungus *Talaromyces flavus* isolated from the leaves of the mangrove plant *Sonneratia apetala*, together with the known compound steperoxide B (merulin A) (**48**) (Figure 4) [18]. Talaperoxides A–D constitute two isomeric pairs. The absolute configuration of the new compounds **44** and **45** together with the known **48** was determined through single crystal X-ray diffraction analysis. Compounds **44**–**48** were investigated for cytotoxic activity against five human cancer cell lines, including HepG2 (liver), HeLa (cervix), PC-3 (prostate), MCF-7 and MDA-MB-435 (breast). Compounds **45** and **47** showed potent cytotoxic activity against the five cell lines being most active against PC-3 cell line with IC_50_ values 3.00 and 2.78 µM, respectively. This variation in activity is most properly due to the *R* configuration at C-7 compared to the *S* configuration for their congeners **44** and **46**, and due to the presence of an acetyl or carbonyl groups at C-3 as compared to steperoxide B (merulin A) (**48**). Therefore, both the *R* configuration at C-7 and the presence of a carbonyl or acetyl groups at C-3 were assumed to be responsible for the potent cytotoxic activity.

Simultaneously, another research group isolated a group of structurally related sesquiterpene endoperoxides including merulin D (**51**) together with the known merulins B (**49**) and C (**50**), and steperoxide A (**52**) from the basidiomycete fungus XG8D obtained from the leaves of the mangrove plant *Xylocarpus granatum* (Figure 4) [19]. Merulin D (steperoxide B) (**51**) was found to be an epimer of **49** differentiated mainly through the 2D NOESY experiment. The antiangiogenic activity for compounds **49**–**52** was investigated revealing that only compounds **50** and **52** possess activity with compound **50** (IC_50_ = 2.5 µM) being ten times more potent than **52** (IC_50_ = 25 µM). These results provided evidence for the possible roles of the C4–C7 endoperoxide linkage as well as for the α,β-unsaturated ketone and hydroxymethyl functionalities. The antiangiogenic activity of compound **50** was further evaluated through a series of *in vitro* and *in vivo* experiments. Compound **50** was found to inhibit neovascularization through suppression of VEGF-induced endothelial cell proliferation and migration via the reduction in Erk1/2 phosphorylation.

Asperaculin A (**53**), a new sesquiterpenoid, was isolated from the sponge derived fungus *Aspergillus aculeatus* CRI323-04 obtained from the sponge *Xestospongia testudinaria* (Figure 4) [20]. Structurally, **53** was revealed to exhibit a new [5,5,5,6] fenestrane sesquiterpenoid ring system, which was given the trivial name aspergillane. The authors proposed a scheme for the biosynthesis of **53** through double bond migration of the known sesquiterpenoid silphinene. Asperaculin A (**53**) was assessed for its antiproliferative activity against HepG2, MOLT-3, A549, and HuCCA-1 cancer cell lines; however, it didn’t exhibit activity up to 50 µg/mL (180 µM) concentration [20].

Four drimane-type sesquiterpenoid lactones (**54**–**57**) were isolated from the fungus *Aspergillus insuetus* (OY-207) isolated from the Mediterranean sponge *Psammocinia* sp. (Figure 4) [21]. The isolated compounds were found to be derivatives of strobilactone A including the new compound, (*E*)-6-(4' hydroxy-2'-butenoyl)-strobilactone A (**54**), the known derivatives strobilactone A (**56**) and (*E*,*E*)-6-(6',7'-dihydroxy-2',4'-octadienoyl)-strobilactone A (**57**) together with 2α,9α,11-trihydroxy-6-oxodrim-7-ene (**55**) [21]. The cytotoxic and antifungal activities of compounds **54**–**57** were investigated and results showed that **54** and **57** exhibited weak cytotoxic activity against MOLT-4 cancer cell lines whereas **56** and **57** showed mild antifungal activity against the fungus *Neurospora crassa*.

Three new bisabolane sesquiterpenoids, aspergillusene A,B and (+)-(7*S*)-7-*O*-methylsydonic acid (**58**–**60**) were isolated from the fungus *Aspergillus sydowii* PSU-F154 isolated from a sea fan, *Annella* sp. (Figure 4) [22]. The isolated compounds were found to be derivatives of the known (+)-sydonic acid (**29**). Compounds **58**–**60** were found inactive in the antioxidant DPPH assay.

A new monocyclic farnesyl sesquiterpene named 3,7,10-trihydroxy-6,11-cyclofarnes-1-ene (**61**) together with a new acorane sesquiterpene, 8-(hydroxymethyl)-1-(2-hydroxy-1-methylethyl)-4-methylspiro[4.5]-dec-8-en-7-ol (**62**), were isolated from the marine-derived fungus *Eutypella scoparia* FS26 isolated from a sediment sample from the South China Sea (Figure 4) [23]. Compounds **61** and **62** were investigated for cytotoxicity against a panel of cancer cell lines but they only showed a weak cytotoxic activity against MCF-7 cell line.

**Figure 5:**
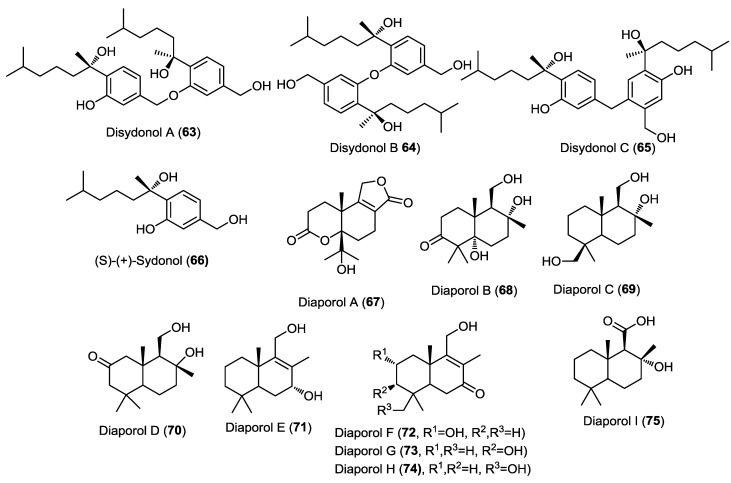
Sesquiterpenes isolated from *Aspergillus* sp. and *Diaporthe* sp. IFB-3lp-10.

Three dimeric bisabolane sesquiterpenoids, disydonols A–C (**63**–**65**), together with the known precursor (+)-*S*-sydonol (**66**), were isolated from the marine-derived fungus *Aspergillus* sp., isolated from the sponge *Xestospongia testudinaria* (Figure 5) [24]. The absolute configuration of the new compounds at C-7 and C-7' was estimated tentatively by comparing their optical rotation to the known precursor *S*-sydonol. This was the second report for the isolation of dimeric bisabolane sesquiterpenes from nature. Disydonol C (**65**) revealed selective *in vitro* cytotoxic activity toward HepG-2 and Caski cancer cell lines with IC_50_ values of 6.0 and 21.0 µM, respectively. However, disydonol A (**63**) displayed moderate cytotoxicity toward these two cell lines with IC_50_ values of 19.1 and 25.5 µM, respectively, where disydonol B (**64**) was found to be relatively noncytotoxic at a concentration up to 200 µM. It was observed that disydonols A (**63**) and C (**65**), possessing the 7*S* and 7'*S* configurations, displayed more potent cytotoxicity toward the tumor cell lines than **64**, with the 7*S* and 7'*R* configurations. These results indicated that the cytotoxic activity might be weakened due to mesomeric effect and thus, the activity of compounds is stereoselective.

Nine new sesquiterpenoids, diaporols A–I (**67**–**75**), were isolated from the endophytic fungus *Diaporthe* sp. IFB-3lp-10 isolated from the leaves of the mangrove *Rhizophora stylosa* (Figure 5) [25]. The parent compound, diaporol A (**67**), constitutes a unique tricyclic lactone sesquiterpene while the other diaporols belong to the drimane type sesquiterpenoids. The absolute configuration of compounds **67**–**71** was determined using single crystal X-ray diffraction. All isolated compounds were subjected to a cytotoxicity assay against a panel of cancer cell lines but none of them showed remarkable activity.

**Figure 6:**
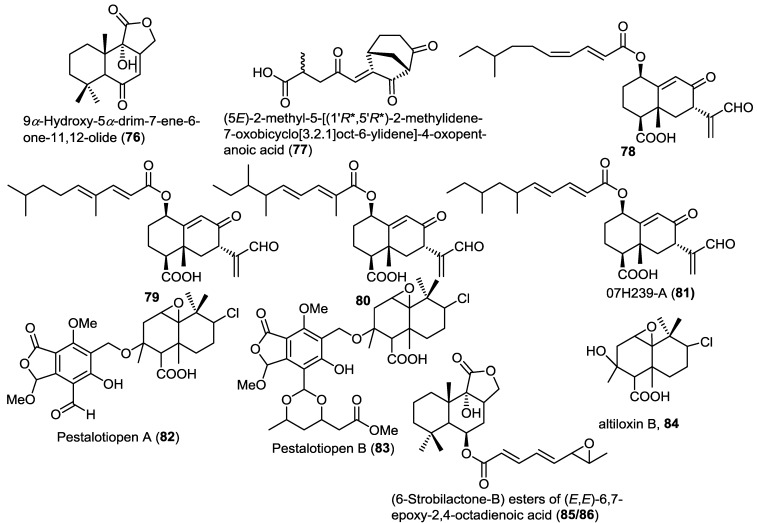
Sesquiterpenes isolated from *Aspergillus carneus* KMM 4638, *Emericellopsis minima*, *Xylaria* sp. BL321, *Pestalotiopsis* sp. and *Aspergillus ustus*.

A new drimane sesquiterpene lactone, 9α-hydroxy-5α-drim-7-ene-6-one-11,12-olide (**76**), was isolated from the endophytic fungus *Aspergillus carneus* KMM 4638, obtained from the marine brown alga *Laminaria sachalinensis* (Figure 6) [26]. Compound **76** is strongly related to the previously reported lactone derivatives such as strobilactone **56**.

A new bicyclic sesquiterpene with unusual bicyclo[3.2.1]octane skeleton, (5*E*)-2-methyl-5-[(1'R*,5'R*)-2-methylidene-7-oxobicyclo[3.2.1]oct-6-ylidene]-4-oxopentanoic acid (**77**), was isolated from the sponge-associated fungus *Emericellopsis minima* obtained from the marine sponge *Hyrtios erecta* (Figure 6) [27]. Compound **77** showed neither cytotoxic nor antimicrobial activities.

Three new eremophilane-type sesquiterpenoids (**78**–**80**), together with the known congener 07H239-A (**81**), were isolated from the mangrove associated fungus *Xylaria* sp. BL321 (Figure 6) [28]. A cytotoxicity assay of compounds **78**–**80** revealed no activity against different cancer cell lines in contrast to the related **81** which was previously reported to possess cytotoxicity [29]. Compound **81** showed dose-dependent activation followed by gradual inhibition of α-glucosidase.

Two new hybrid drimane sesquiterpene-cyclopaldic acids, pestalotiopens A,B (**82** and **83**), were isolated from the mangrove derived fungus *Pestalotiopsis* sp. obtained from leaves of the Chinese mangrove *Rhizophora mucronata* (Figure 6) [30]. Pestalotiopens were identified as ethers of the known altiloxin B (**84**), which was also isolated in the same report with cyclopaldic acid. Compound **83** possesses an extra triketide moiety. The authors proposed a biogenetic pathway for the new compounds involving altiloxin B as a common precursor. The antibacterial activity of the new compounds was assessed. Pestalotiopen A (**82**) showed a moderate activity against *Enterococcus faecalis*.

Further investigation of the fungus *Aspergillus ustus* obtained from the marine alga *Codium fragile* resulted in the isolation of two new isomeric strobilactone B esters of (*E*,*E*)-6,7-epoxy-2,4-octadienoic acid (**85** and **86**) (Figure 6) [31]. The new compounds displayed potent lethal activity against brine shrimp.

**Figure 7:**
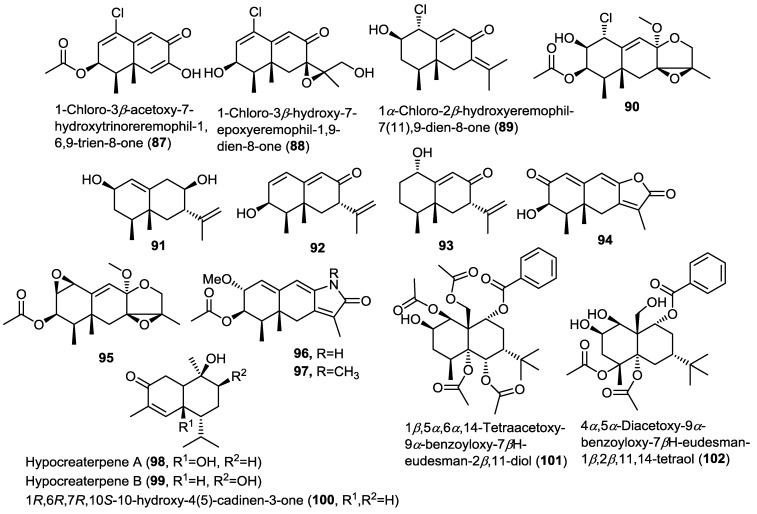
Sesquiterpenes isolated from *Penicillium* sp. PR19N-1, *Hypocreales* sp. HLS-104 and *Pestalotiopsis* sp.

Four new chlorinated eremophilane-type sesquiterpenoids were isolated from the fungus *Penicillium sp.* PR19N-1 obtained from an Antarctic deep sea marine sludge [32]. The new compounds were identified as 1-chloro-3β-acetoxy-7-hydroxytrinoreremophil-1,6,9-trien-8-one (**87**), 1-chloro-3β-hydroxy-7-epoxyeremophil-1,9-dien-8-one (**88**), 1α-chloro-2β-hydroxyeremophil-7(11),9-dien-8-one (**89**), and epoxy-tetrahydrofuran eremophilane (**90**). The authors proposed a biosynthetic pathway for compounds **87**–**90** involving biochemical modifications of the key intermediate 1α-hydroxy-7βH-eremophil-9,11-dien-8-one (**93**), that was subsequently reported by the same group in another study (Figure 7). Compounds **87**–**90** were evaluated for their cytotoxic activity against HL-60 and A549 cancer cells. Only **87** showed moderate cytotoxicity.

The same research group conducted further investigation on the same fungal material leading to the isolation of six additional new eremophilane sesquiterpenoids (**91**–**96**) (Figure 7) [33]. Compound **96** belongs to the rare eremophilane lactam-type that is strongly related to the semi-synthetic compound **97** [31]. New compounds were evaluated for their cytotoxic activity against HL-60 and A549 cell lines but only compounds **91** and **95** were found to possess moderate cytotoxicity.

Investigation of the fungus *Hypocreales* sp. HLS-104 isolated from the marine sponge *Gelliodes carnosa* led to the isolation of two new cadinane-type sesquiterpenoids hypocreaterpenes A and B (**98** and **99**) along with the known 1*R*,6*R*,7*R*,10*S*-10-hydroxy-4(5)-cadinen-3-one (**100**) (Figure 7) [34]. HPLC chromatograms of the extracts obtained from fungal cultures prepared with distilled water and with sea water were compared revealing that sea water based media were superior with regard to the induction of natural products accumulation. Compounds **98**–**100** were tested for their anti-inflammatory activity via estimating the inhibitory activity on nitric oxide production in RAW 264.7 cell assay. Only **100** showed moderate inhibition, highlighting the possible role of the 6- or 9-hydroxyl group in decreasing or inhibiting nitric oxide production.

A report on the endophytic fungus *Pestalotiopsis* sp. isolated from the marine alga *Sargassum horneri* showed the isolation of two new highly esterified sesquiterpenoids 1β,5α,6α,14-tetraacetoxy-9α-benzoyloxy-7βH-eudesman-2β,11-diol (**101**) and 4α,5α-diacetoxy-9α-benzoyloxy-7βH-eudesman-1β,2β,11,14-tetraol (**102**) (Figure 7) [35]. Compounds **101** and **102** were produced as a result of abiotic stress on the fungus in the culture media using CuCl_2_ as revealed by comparison of the TLC chromatograms of stressed and non-stressed fungal cultures. Tyrosinase inhibitory activity of both **101** and **102** was evaluated revealing their potent enzyme inhibitory activity with IC_50_ values of 14.8 and 22.3 µM, respectively. These results point to the possibility of their progress as future candidates for clinical trials in hyperpigmentation conditions.

**Figure 8:**
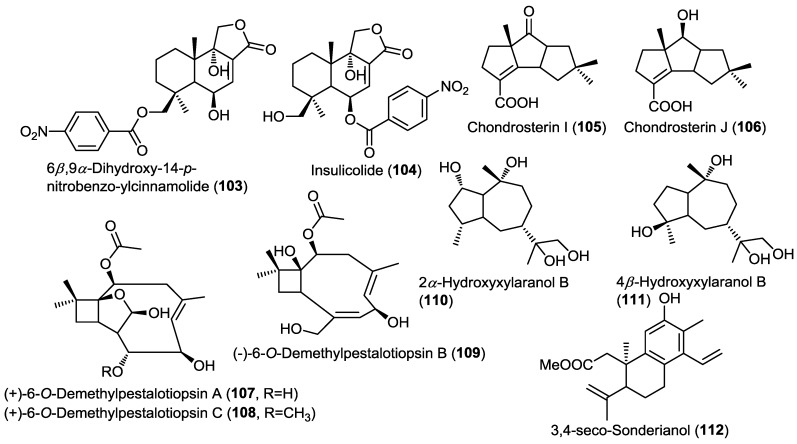
Sesquiterpenes isolated from *Aspergillus ochraceus* Jcma1F17, *Chondrostereum* sp., *Ascotricha* sp. ZJ-M-5 and endophytic fungus J3.

Investigation of the endophytic fungus *Aspergillus ochraceus* Jcma1F17 isolated from the marine alga *Coelarthrum* sp. led to the isolation of the new nitrobenzoyl sesquiterpene 6β,9α-dihydroxy-14-*p*-nitrobenzoylcinnamolide (**103**) and the known positional isomer insulicolide A (**104**) (Figure 8) [36]. Nitrobenzoyl sesquiterpenoids are very rare natural compounds and to the best of our knowledge they are restricted to *Aspergillus* sp. and considered as chemotaxonomic markers for few *Aspergillus* species.

Compounds **103** and **104** showed potent cytotoxic activity against a panel of 10 human cancer cell lines (H1975, U937, K562, BCG-823, Molt-4, MCF-7, A549, HeLa, HL60, and Huh-7), with IC_50_ values of 1.95 to 6.35 µM. In addition, **103** showed potent antiviral activity against influenza virus H_3_N_2_ and human enterovirus EV71 with IC_50_ values of 17.0 and 9.4 µM, respectively [36].

Two new unique hirsutane sesquiterpenoids were isolated from the fungus *Chondrostereum* sp. isolated from the soft coral *Sarcophyton tortuosum* (Figure 8) [37]. The new compounds were identified as chondrosterin I (**105**) and J (**106**) based on 1D, 2D NMR, MS and single crystal X-ray diffraction [37]. The authors proposed that changing the carbon source in the culture media from glucose to glycerol was the main reason for the dramatic changes in the hirsutane nucleus observed in the new compounds when compared to their previously reported congeners isolated from the same fungus, with a migration of C-2 methyl to C-6 and the introduction of carboxyl group at C-3. This experimental approach of changing the components of culture media in order to trigger the stimulation or inhibition of certain genes and subsequently changing the secondary metabolites profile is a well-known technique called One Strain Many Compounds Analysis (OSMAC). Compound **106** showed potent cytotoxic activity against human nasopharyngeal cancer cell lines CNE-1 and 2 with IC_50_ values of 1.32 and 0.56 µM, respectively; whereas **105** was found to be inactive. These results point out to the importance of the 7-OH group for the activity of the compound.

The OSMAC approach was also employed by another research group by changing the MgCl_2_ concentration in the culture media of the fungus *Ascotricha s*p. ZJ-M-5 that had been obtained from a mud sample collected on the coastline of Fenghua, China [38]. Modifications of the Czapek Dox broth culture media by adding different concentrations of MgCl_2_ or completely removing it resulted in the isolation of three new caryophyllene-type sesquiterpenes, (+)-6-*O*-demethylpestalotiopsin A and C together with (−)-6-*O*-demethylpestalotiopsin B (**107**–**109**) (Figure 8) [38]. Compounds **107** and **108** were produced in response to the absence of MgCl_2_ in the culture media whereas the addition of Mg^2+^ resulted in suppression of their production but stimulated the production of **109**. Further increase of the Mg^2+^ concentration in the broth inhibited the production of compounds **107**–**109**. Compounds **107** and **108** showed potent growth inhibitory activity against HL-60 and K562 with IC_50_ values ranging between 6.9 and 12.3 µM; while **109** showed no activity at all.

Two new sesquiterpenoids, 2α-hydroxyxylaranol B (**110**) and 4β-hydroxyxylaranol B (**111**), together with the known diterpene 3,4-seco-sonderianol (**112**) were isolated from the endophytic fungus J3 obtained from leaves of the mangrove *Ceriops tagal* (Figure 8) [39]. Evaluation of the cytotoxic activities of compounds **110**–**112** against K562, SGC-7901, and BEL-7402 cancer cell lines revealed that compound **112** exhibited moderate cytotoxic activity against all the three cell lines whereas the new compounds did not show any cytotoxic activity.

### 2.3. Diterpenes (C20)

**Figure 9:**
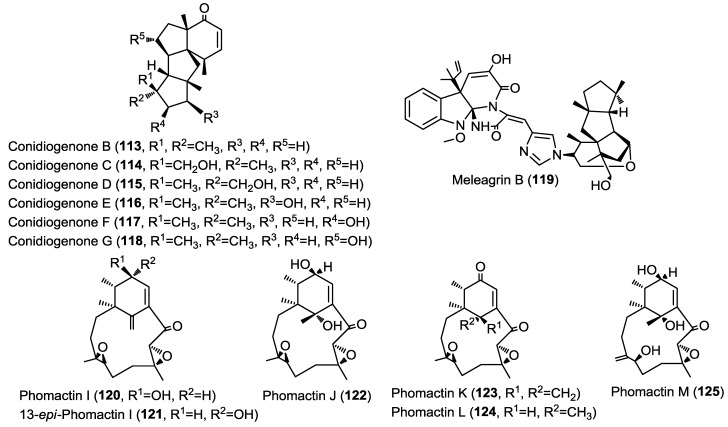
Diterpenes isolated from *Penicillium* sp., unidentified fungal strain (MPUC 046) and *Phoma* sp.

Six new diterpenes were isolated from the marine-derived fungus *Penicillium* sp. strain F23-2 cultured from deep ocean sediment. The new diterpenes were found to belong to the very rare conidiogenone class and were identified as conidiogenone B–G (**113**–**118**) (Figure 9) [40]. Conidiogenone G (**118**) was postulated by the authors as a biosynthetic precursor for the new diterpene alkaloid meleagrin B (**119**) co-isolated in the same report. The proposed biosynthetic scheme comprises several steps with a Michael addition reaction as a key reaction. Compounds **113**–**119** were evaluated for their cytotoxic activity against HL-60, A549, BEL-7402 and MOLT-4 cancer cell lines. Conidiogenone C (**114**) showed potent cytotoxicity against HL-60 and BEL-7402 cells with IC_50_ values of 0.038 and 0.97 µM whereas meleagrin B (**119**) showed moderate cytotoxicity against all cell lines investigated.

Three new macrocyclic epoxy-diterpenes were isolated from an unidentified fungal strain (MPUC 046) obtained from the marine brown alga *Ishige okamurae* [41]. The isolated compounds are similar to the known platelet activating factor (PAF) antagonists, phomactins and were identified as phomactin I, 13-*epi*-phomactin I and phomactin J (**120**–**122**) (Figure 9) [41]. The absolute configuration was assessed with the aid of the single crystal X-ray diffraction experiment. The same authors had previously reported the unique phomactin H from the same fungal material and it is worth noting that the earlier members of this series were isolated from a phylogenetically different fungal species, *Phoma* sp. [42].

Further investigation of the unidentified fungal strain (MPUC 046) obtained from the marine brown alga *Ishige okamurae* yielded three macrocyclic diterpenes, phomactins K–M (**123**–**125**) (Figure 9) [43]. Identification of the isolated compounds was achieved using single X-ray crystal diffraction analysis and by comparing the obtained spectra with the previously reported data of related congeners. It is worth noting that phomactins were found to possess a significant platelet activating factor inhibitory effect. Additionally, most of these compounds were inactive in several other biological assays suggesting their high selectivity towards PAF inhibitory effect and their potential as future candidates for clinical trials.

**Figure 10:**
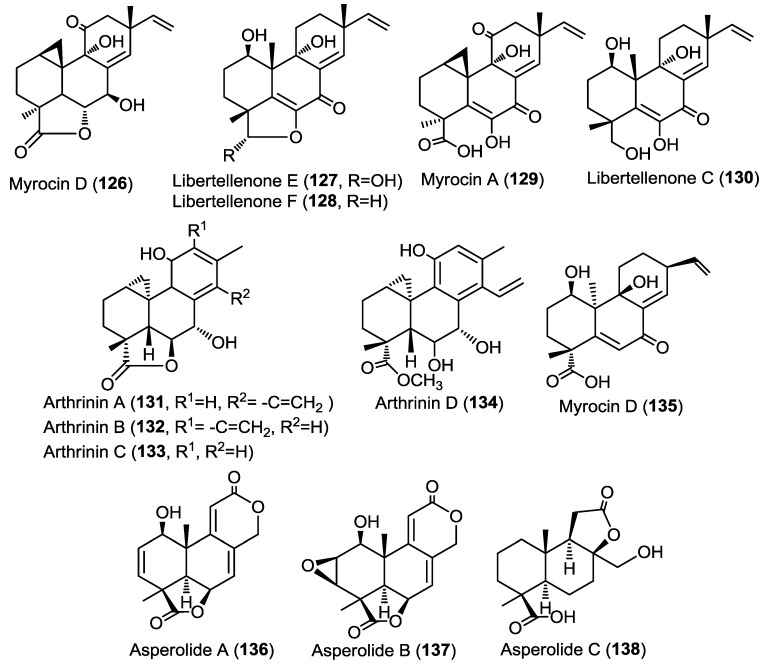
Diterpenes isolated from *Arthrinium sacchari*, *Libertella* sp., *Arthrinium* sp. and *Aspergillus wentii* EN-48.

The same research group had reported the isolation of three new diterpenes myrocin D, libertellenone E and F (**126**–**128**), together with the known congeners myrocin A (**129**) and libertellenone C (**130**), from the fungus *Arthrinium sacchari* obtained from an unidentified sponge collected from the coast of Atami-shi (Figure 10) [44]. The absolute configuration of the new compounds was determined using single crystal X-ray diffraction analysis.

Earlier members of the libertellenone series, libertellenone A–D, were previously reported from the fungus *Libertella* sp. as a response to an induced stress caused by co-culturing the fungus with a marine α-proteobacterium (strain CNJ-328) [45]. Compounds **126**–**130** were evaluated for their antiangiogenic activity through measuring their ability to inhibit the proliferation of human umbilical vein endothelial cells (HUVECs) and human umbilical artery endothelial cells (HUAECs) (Figure 10). Only **129** and **130** showed inhibitory activity whereas the other compounds were inactive.

In a parallel study on the marine fungus *Arthrinium* sp. derived from the Mediterranean sponge *Geodia cydonium*, four novel diterpenoids, arthrinins A–D (**131**–**134**), were identified from its methanol extract. In addition, one new diterpenoid, myrocin D (**135**) along with five known compounds including myrocin A and two xanthone derivatives, norlichexanthone and anomalin A were purified from the same extract (Figure 10) [46]. The structures of arthrinins A–D (**131**–**134**) were recognized as being of hybrid origin and derived from cleistanthane and pimarane diterpenes. The absolute configuration of arthrinins A–D (**131**–**134**) was established by the modified Mosher’s method and by ROESY spectra. Antiproliferative activity of the isolated compounds was evaluated toward four different tumor cell lines, namely, L5178Y, K562, A2780, and A2780CisR cell lines. Results revealed that norlichexanthone and anomalin featured the strongest activities (IC_50_ values of 0.40–74.0 µM) [46]. These findings are in accordance with results from protein kinase activity assays that included aurora-B, PIM-1, and VEGF-R2 kinases which were inhibited by norlichexanthone and anomalin A with IC_50_ values between 0.3 and 11.7 µM [46]. Furthermore, in the *in vitro* angiogenesis assay against HUVECs sprouting induced by VEGF-A, myrocins D (**135**), A, and anomalin A inhibited endothelial cell sprouting with IC_50_ values of 2.6, 3.7, and 1.8 µM, respectively.

**Figure 11:**
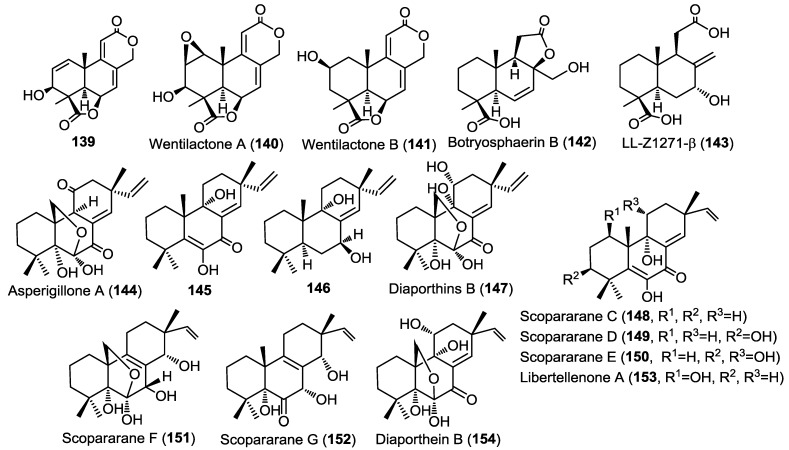
Diterpenes isolated from *Aspergillus wentii* EN-48, fungal strain HS-1, *Eutypella scoparia* FS26.

Three new tetranorlabdane diterpenoids, asperolides A–C (**136**–**138**) (Figure 10), together with five known derivatives, a tetranorditerpenoid derivative (**139**), wentilactones A (**140**) and B (**141**), botryosphaerin B (**142**) and LL-Z1271-β (**143**) (Figure 11) were isolated from the endophytic fungus *Aspergillus wentii* EN-48 cultured from the marine algae *Sargassum* sp. [47]. All isolated compounds were evaluated for their cytotoxic and antimicrobial activities against a panel of cancer cell lines and microbial strains, respectively. Compounds **136**, **137**, and **139**–**141** showed moderate cytotoxicity with wentilactone B (**141**) as the most potent among the tested compounds (IC_50_ = 17 µM). In the antimicrobial assay, compound **139** showed considerable antifungal activity against *Candida albicans* with an MIC value of 55.6 µM [47].

Three new pimarane diterpenoids (**144**–**146**) together with the known diaporthins B (**147**) were isolated from the fungal strain HS-1 cultured from the sea cucumber *Apostichopus japonicus* (Figure 11) [48]. Diaporthins B (**147**) was used as a reference in the determination of the absolute configuration of the new compounds via comparison of the circular dichroism (CD) spectra.

All isolated compounds were evaluated for their cytotoxic activities against KB and KBv200 cell lines. Compounds **144** and **147** showed potent activity against both cell lines with IC_50_ of 10.1, 6.8 µM and 10.6, 17.9 µM, respectively. Compound **145** was only weakly active (IC_50_ > 45 µM) whereas compound **146** showed no activity, pointing out to the possible role of carbonyl group at C-7 in the cytotoxic activity.

Five new pimarane diterpenoids, scopararanes C–G (**148**–**152**), together with six known pimarane diterpenes, were isolated from the marine-derived fungus *Eutypella scoparia* FS26 that had been obtained from sediment collected in the South China Sea (Figure 11) [49]. All isolated compounds were assessed for their antiproliferative activity using a cytotoxicity (MTT) assay against three different human cancer cell lines; including MCF-7 (breast), NCI-H460 (lung), and SF-268 (brain). Among the tested compounds, scopararane D (**149**) showed only mild antiproliferative activity with IC_50_ values between 25.6 and 46.0 µM, whereas, the known compounds, libertellenone A (**153**) and diaporthein B (**154**), revealed potent antiproliferative activities with IC_50_ values ranging from 4.4–20.0 µM, compared to cisplatin (IC_50_ = 1.5–9.2 µM) (Figure 11) [49].

### 2.4. Sesterterpenes (C25)

**Figure 12:**
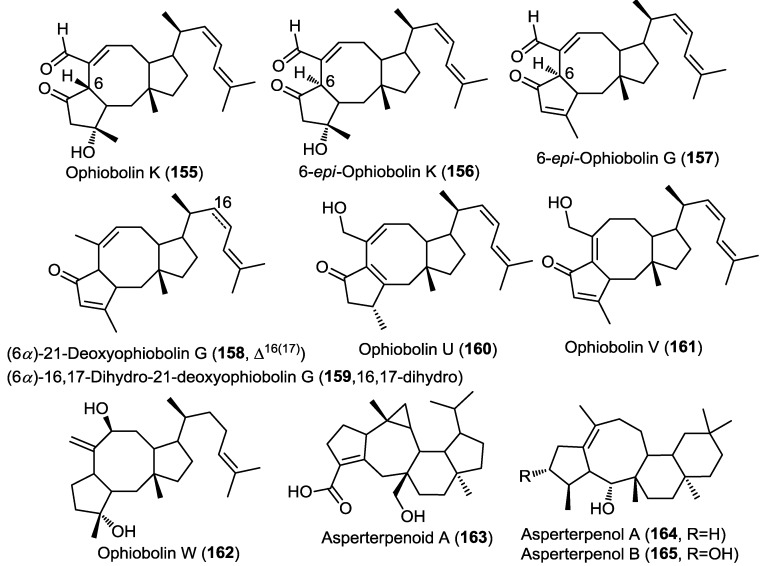
Sesterterpenes isolated from *Emericella variecolor* strain GF-10, *Aspergillus ustus*, *Aspergillus* sp. 16-5C and *Aspergillus* sp. 085242.

Three new sesterterpenes belonging to the ophiobolin class were isolated from the marine derived fungus *Emericella variecolor* strain GF-10 obtained from sediment collected at 70 m depth from the Gokasyo Gulf, Japan [50]. The isolated compounds were identified as ophiobolin K, 6-*epi*-ophiobolin K and 6-*epi*-ophiobolin G (**155**–**157**) (Figure 12) [50] and were found to inhibit biofilm formation of *Mycobacterium smegmatis* with compound **155** being the most active (MIC = 4.1 µM). However, isolated compounds failed to show remarkable antimicrobial activity. 6-*epi*-Ophiobolin G (**157**) was further studied using *M. bovis* BCG and found to inhibit its biofilm formation at a MIC = 8.2 µM [50]. Moreover, the compound showed the ability to restore the antimycobacterial effect of isoniazid against biofilm forming *M. smegmatis* [50].

Five ophiobolin sesterterpenes were obtained from the fungus *Aspergillus ustus* isolated from the marine alga *Codium fragile* [31]. The compounds were identified as (6α)-21-deoxyophiobolin G, (6α)-16,17-dihydro-21-deoxyophiobolin G, and ophiobolins U–W (**158**–**162**) (Figure 12) [31]. All compounds were evaluated for their antibacterial and antifungal activity and for their lethal effect on brine shrimp eggs. Only compound **160** showed a remarkable antibacterial activity against *Escherichia coli* and *Staphylococcus aureus* as well as >75% lethality in the brine shrimp toxicity assay [31].

Investigation of the mangrove-derived fungus *Aspergillus sp.* 16-5C resulted in the isolation of a new pentacyclic sesterterpene, asperterpenoid A (**163**) (Figure 12) [51]. The absolute configuration of **163** was determined with the X-ray single crystal diffraction analysis. Compound **163** was found to possess an unprecedented pentacyclic nucleus; the authors had proposed a biosynthetic scheme involving an initial geranyl farnesyl pyrophosphate (GFPP) moiety undergoing a series of cyclization, group migration and oxidation reactions. Asperterpenoid A (**163**) showed potent inhibitory activity against *Mycobacterium tuberculosis* protein tyrosine phosphatase B (mPTPB) with IC_50_ = 2.2 µM [51].

The same authors reported two additional new tetracyclic sesterterpenes, asperterpenol A (**164**) and B (**165**) from the mangrove-derived fungus *Aspergillus* sp. 085242 (Figure 12) [52]. The new compounds were found to possess an unprecedented 5/8/6/6 tetracarbocyclic nucleus; similarly the authors proposed a biosynthetic scheme for **164** and **165** involving a geranyl farnesyl pyrophosphate (GFPP) substrate undergoing a series of cyclization, group migration and oxidation reactions. Compounds **164** and **165** were evaluated for their anticholinesterase activity against acetylcholinesterase (AChE) and butyrylcholinesterase enzymes (BuChE). Both compounds showed potent inhibition of AChE at IC_50_ values of 2.3 and 3.0 µM, respectively. However, neither of them showed inhibition of BuChE, pointing to a selective activity.

### 2.5. Triterpenes (C30)

**Figure 13:**
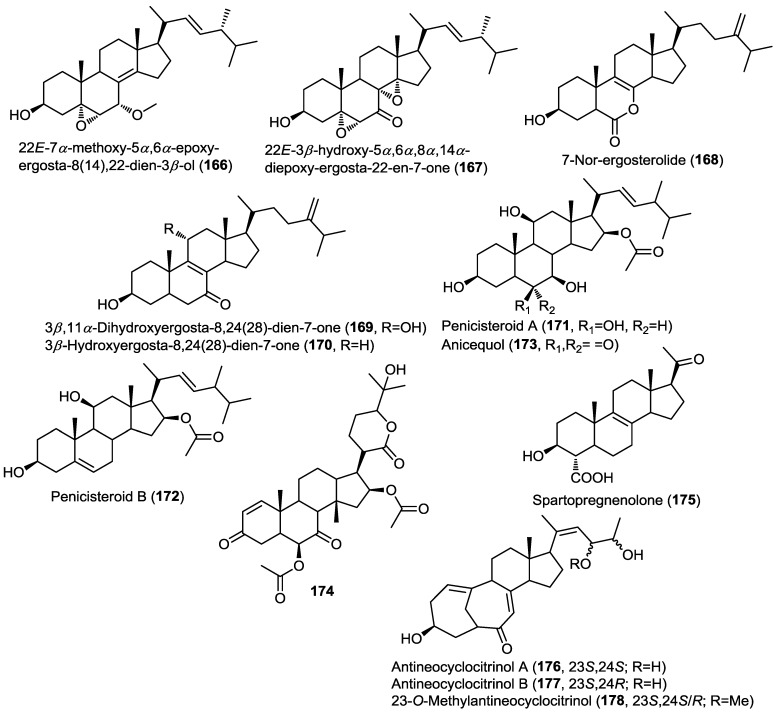
Triterpenes isolated from *Aspergillus awamori*, *Aspergillus ochraceus* EN-31, *Penicillium chrysogenum* QEN-24s, *Aspergillus fumigatus* KMM4631, *Phaeosphaeria spartinae* and mutant AD-1-2 *Penicillium purpurogenum* G59.

Two new oxidized ergosterols, 22E-7α-methoxy-5α,6α-epoxy-ergosta-8(14),22-dien-3β-ol (**166**) and 22E-3β-hydroxy-5α,6α,8α,14α-diepoxy-ergosta-22-en-7-one (**167**) were isolated from the fungus *Aspergillus awamori* obtained from soil surrounding the mangrove plant *Acrostichum speciosum* (Figure 13) [53]. Analysis of the ROESY spectrum of **166** showed that the endo-boat conformation of ring B is the most reasonable conformation instead of the half-chair conformation generally adopted for oxidized cyclohexene ring systems. It was suggested that this conformation was adopted due to the stabilization effect of C8/C14 double bond. Compounds **166** and **167** exhibited weak cytotoxic activity against A549 cancer cell lines.

Three ergosterol derivatives were isolated from the fungus *Aspergillus ochraceus* EN-31 obtained from the marine brown alga *Sargassum kjellmanianum* [54]. The isolated compounds were identified as 7-nor-ergosterolide (**168**), 3β,11α-dihydroxyergosta-8,24(28)-dien-7-one (**169**) and 3β-hydroxyergosta-8,24(28)-dien-7-one (**170**) (Figure 13) [54]. The absolute configuration of the isolated compounds was determined by the modified Mosher’s method. Compound **168** possesses an unprecedented pentalactone B-ring system. The presence of this unusual lactone moiety in ring B was suggested on the basis of a missing signal from the usual 28 signals of ergosterol as revealed by the ^13^C spectra and the structure was confirmed through HMBC correlations. The authors proposed a biosynthetic scheme for the unusual compound **168** involving the co-isolated **170** as a precursor through the ring-B opening, decarboxylation, oxidation and final intermolecular esterification. Compounds **168**–**170** were evaluated for their cytotoxicity as well as for their antibacterial and antifungal activities. Compound **168** showed selective cytotoxicity against NCI-H460, SMMC-7721, and SW1990 cell lines, whereas **169** showed activity against SMMC-7721 cell line. However, none of the tested compounds showed any antimicrobial activity.

Investigation of the endophytic fungus *Penicillium chrysogenum* QEN-24s, isolated from the marine red alga *Laurenica* sp., led to the isolation of two polyoxygenated new sterols, penicisteroid A (**171**) and B (**172**), together with the previously reported steroid, anicequol (**173**) (Figure 13) [55]. Compounds **171**–**173** shared the unique structural feature of having 11-OH and 16-acetoxy functionalities; to the best of our knowledge no other natural products possess this feature. The isolated compounds were subjected to cytotoxic and antifungal evaluation. Results disclosed that penicisteroid A (**171**) showed antifungal activity against both *Aspergillus niger* and *Alternaria brassicae* whereas anicequol (**173**) showed activity only against *A. brassicae* while penicisteroid B (**172**) did not exhibit activity against the two fungi. This may point to the role of the 6-OH function in the activity against *A. niger* and the role of OH-substitution in ring B for the activity against *A. brassicae*. Similarly, **171** exhibited cytotoxicity against a panel of cell lines HeLa, SW1990, and NCI-H460 (IC_50_ values of 29.6–79.0 µM) with no activity observed for the other compounds.

6β,16β-Diacetoxy-25-hydroxy-3,7-dioxo-29-nordammara-1,17(20)-dien-21,24-lactone (**174**), a new nor-dammarane triterpene, was isolated from the fungus *Aspergillus fumigatus* KMM4631 obtained from the soft coral *Sinularia* sp. (Figure 13) [56]. The isolated compound was evaluated for its antibacterial and antifungal activities but did not show any remarkable activity.

Spartopregnenolone (**175**), a new unique pregnane-type sterol, was isolated from the marine derived endophytic fungus *Phaeosphaeria spartinae* cultured from the marine red *alga Ceramium sp.* (Figure 13) [57]. Spartopregnenolone shares structural features common to lanosterol triterpenes, mainly the presence of a ∆^8,9^ double bond, whereas the other features are more common to pregnane-type sterols, mainly the presence of 17-acetyl side chain instead of the usual isoprene unit. The authors argued that **175** is an intermediate in the biosynthesis of sterols from lanosterol, the presence of the unusual 4-carboxylic group confirmed this assumption.

Three new steroids, antineocyclocitrinols A, B and 23-*O*-methylantineocyclocitrinol (**176**–**178**), were isolated from the mutant AD-1-2. This mutant was obtained by mutagenesis of the fungal strain *Penicillium purpurogenum* G59 collected from a soil sample at the tideland of Bohai Bay, China (Figure 13) [58]. LC/MS and HPLC-PDA chromatograms of the mutant strain revealed the presence of metabolites not detectable in the original extract of G59. Moreover, the ethyl acetate extract of AD-1-2 showed significant cytotoxicity whereas the wild type did not. Mutagenesis is a well-established technique for activation of silent genes and thus the production of unusual metabolites. In this report, the authors used the known diethyl sulfate (DES) as a mutagen. The isolated sterols shared the unusual bicyclo[4.4.1]A/B ring system and showed weak cytotoxicity against a panel of cancer cell lines, including K562, HL-60 and HeLa cells.

### 2.6. Meroterpenes

**Figure 14:**
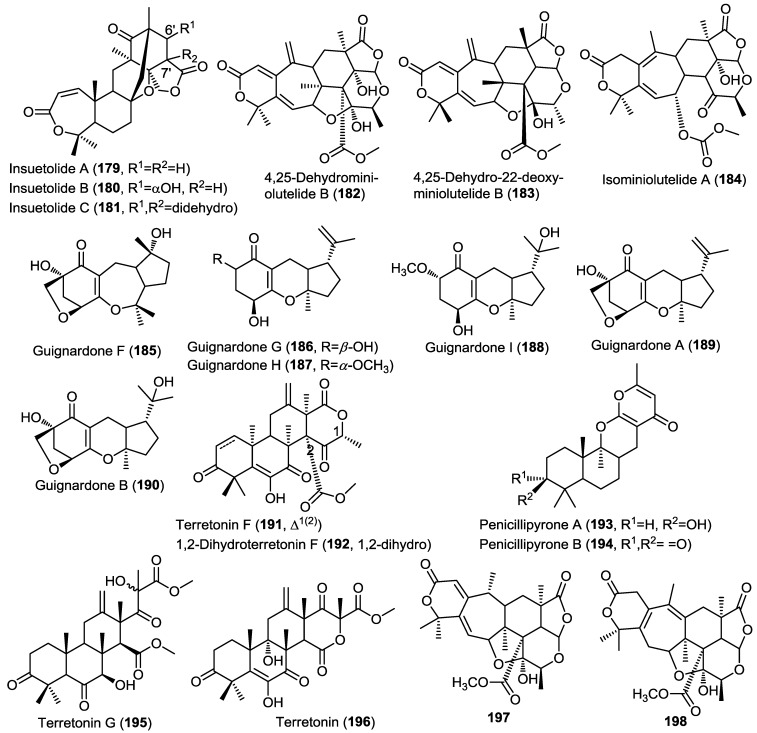
Meroterpenes isolated from *Aspergillus insuetus* (OY-207), *Penicillium* sp. MA-37, fungus A1, *Aspergillus ustus*, *Penicillium* sp. F446, *Aspergillus* sp. OPMF00272 and *Penicillium* sp. 303.

Three new meroterpenoids were obtained from the fungus *Aspergillus insuetus* (OY-207) isolated from the Mediterranean sponge *Psammocinia* sp. [21]. The isolated compounds were identified as insuetolides A–C (**179**–**181**) (Figure 14) [21]. Insuetolides constitute a new carbon skeleton in which a drimane sesquiterpene lactone is linked with tetraketide-3,5-dimethylorsellinic acid. The main difference between the insuetolides and their common congeners isolated from marine-derived fungi lies in the uncommon presence of perhydropyran ring C instead of the common cyclopentane ring. The authors pointed out to the possible additional oxidation step in the sesquiterpenoid prior to coupling of C-12 to the tetraketide moiety orsellinic acid. Compound **179** showed antifungal activity against the fungus *Neurospora crassa*, whereas compound **181** showed moderate cytotoxicity against MOLT-4 cancer cell lines.

Three new meroterpenoids were isolated from the fermentation broth of the fungus *Penicillium* sp. MA-37 obtained from sediment close to the mangrove plant *Bruguiera gymnorrhiza* [59]. The compounds were found to be derivatives of miniolutelide B and were identified as 4,25-dehydro-miniolutelide B (**182**), 4,25-dehydro-22-deoxy-miniolutelide B (**183**) and isominiolutelide A (**184**) (Figure 14) [59]. It is worth noting that the organism yielded a completely different class of compounds upon shaking of the cultures belonging to diphenyl ether derivatives including three new congeners, namely, ∆^1^^',3^^'^-1'-dehydropenicillide, 7-*O*-acetylsecopenicillide C, and hydroxytenellic acid B.

Investigation of the fungus A1 isolated from the mangrove plant *Scyphiphora hydrophyllacea* led to the isolation of six structurally related meroterpenoids sharing a guignardone nucleus. The isolated compounds comprised four new members, guignardone F–I (**185**–**188**) together with the previously reported congeners, guignardone A (**189**) and B (**190**) (Figure 14) [60]. The authors proposed a biosynthetic scheme involving compound **189** as a common precursor. All isolated compounds were evaluated for their antibacterial activity against MRSA and *Staphylococcus aureus*. Only compounds **188** and **189** showed activity.

The fungus *Aspergillus ustus* obtained from the marine alga *Codium fragile* yielded two related meroterpenes including the structurally revised terretonin F (**191**) and the new derivative 1,2-dihydroterretonin F (**192**) (Figure 14) [31]. Compound **191** was previously reported but in this study the authors revised its structure and confirmed the presence of a lactone moiety not included in the original structure. Compounds **191** and **192** showed toxicity to brine shrimp (>75% lethality at 49.6 and 138.6 µM, respectively).

Penicillipyrones A (**193**) and B (**194**) are two new meroterpenes isolated from the fungus *Penicillium* sp. F446 obtained from a 25 m deep marine sediment collected from Geomun-do island, Korea (Figure 14) [61]. The new compounds constituted an unprecedented sesquiterpenoid-γ-pyrone skeleton that differs from the previously reported prenylated γ-pyrones in both the linkage and orientation of the pyrone moiety relative to the sesquiterpenoid. In the isolated compounds, the sesquiterpene is connected to the pyrone through C-10 instead of the common C-12 connectivity. Compounds **193** and **194** did not show any remarkable cytotoxic or antibacterial activity, however, **194** showed dosage-dependent induction of quinone reductase in Hepa 1c1c7 cells which may play an important role as a chemopreventive agent.

Investigation of the sponge-derived fungus *Aspergillus* sp. OPMF00272, Japan, resulted in the isolation of an additional terretonin-type meroterpenes, terretonin G (**195**), together with the known terretonin (**196**) (Figure 14) [62]. Compound **195** showed potent antibacterial activity against gram-positive bacteria, however, it did not show activity against gram negative bacteria or fungi, whereas the parent compound, terretonin (**196**) did not show antibacterial or antifungal activity.

Two new meroterpenes (**197** and **198**) were isolated from the marine fungus *Penicillium* sp. 303 cultured from sea water sampled from Zhanjiang Mangrove National Nature Reserve in Guangdong Province, China (Figure 14) [63]. The isolated compounds are structurally related to the miniolutelide class of meroterpenoids and were identified as derivatives of miniolutelide B. Compounds **197** and **198** showed moderate cytotoxic activity against a panel of cancer cell lines including MDA-MB-435 (breast), HepG2 (liver), HCT-116 (colon) and A549 (lung) cells.

## 3. Conclusions

The isolation of terpenoids from marine-derived fungi with unique structures and interesting biological activity continued over the last five years with fascinating results. Different monoterpenes, sesquiterpenes, diterpenes, sesterterpenes, triterpenes and meroterpenes were identified. Based on the summarized reports, a large library of sesquiterpenes was isolated possessing unique structures and potent biological activities including drimane, endoperoxides, nitrobenzoyl and bisabolane sesquiterpenes. Interestingly, changing the growth medium composition had a significant effect on the structures of the isolated sesquiterpenoids and the application of OSMAC strategy led to the isolation of novel compounds. Interesting diterpenes were also isolated such as the rare conidiogenone-type diterpenes and the potent inhibitor of platelet activating factor, phomactins. The conidiogenone-type exhibited the most potent cytotoxic activity against a large panel of cancer cell lines with conidiogenone C as the most potent congener showing IC_50_ values of 0.038 and 0.97 µM against HL-60 and BEL-7402 cells, respectively.

Unique triterpenes with polyoxygenated skeleton as well as 11-OH and 16-acetoxy functionalities were identified and found to possess potent antifungal activity. Meroterpenes with a unique sesquiterpenoid-γ-pyrone skeleton were purified and their chemopreventive activity represents an interesting topic for future research. The richness of marine-derived fungi with diverse terpenoids the feasibility of these organisms to be cultivated on different growth media offer a rare opportunity to expand the natural product chemical space with novel compounds possessing unprecedented chemical structures and biological activities.
